# Estrogen receptor α mediates the effects of notoginsenoside R1 on endotoxin-induced inflammatory and apoptotic responses in H9c2 cardiomyocytes

**DOI:** 10.3892/mmr.2015.3394

**Published:** 2015-02-27

**Authors:** LEI ZHONG, XING-LU ZHOU, YAN-SONG LIU, YI-MIN WANG, FEI MA, BAO-LIANG GUO, ZHAO-QI YAN, QING-YUAN ZHANG

**Affiliations:** 1 Department of General Surgery, Second Affiliated Hospital of Harbin Medical University, Harbin, Heilongjiang 150086, P.R. China; 2 Department of Internal Medicine, Cancer Hospital Affiliated to Harbin Medical University, Harbin, Heilongjiang 150040, P.R. China

**Keywords:** notoginsenoside R1, estrogen receptor, endotoxin, cardiomyocyte

## Abstract

Estrogen receptors (ERs) are important for preventing endotoxin-induced myocardial dysfunction. Therefore, plant-derived phytoestrogens, which target ERs may also affect endotoxin-induced toxicity in cardiomyocytes. Our previous study revealed that notoginsenoside-R1 (NG-R1), a predominant phytoestrogen from *Panax notoginseng*, protects against cardiac dysfunction. However, the effects of NG-R1 on cardiomyocytes and the precise cellular/molecular mechanisms underlying its action remain to be elucidated. In the present study, pretreatment with NG-R1 suppressed the lipopolysaccharide (LPS)-induced degradation of inhibitor of nuclear factor-κB (NF-κB) α, the activation of NF-κB and caspase-3, and the subsequent myocardial inflammatory and apoptotic responses in H9c2 cardiomyocytes. An increase in the mRNA and protein expression of ERα was also observed in the NG-R1-treated cardiomyocytes. However, the expression pattern of ERβ remained unaltered. Furthermore, the cardioprotective properties of NG-R1 against LPS-induced apoptosis and the inflammatory response in cardiomyocytes were attenuated by ICI 182780, a non-selective ERα antagonist, and methyl-piperidino-pyrazole, a selective ERα antagonist. These findings suggested that NG-R1 reduced endotoxin-induced cardiomyocyte apoptosis and the inflammatory response via the activation of ERα. Therefore, NG-R1 exerted direct anti-inflammatory and anti-apoptotic effects on the cardiomyocytes, representing a potent agent for the treatment of myocardial inflammation during septic shock.

## Introduction

The incidence of severe sepsis is rising annually, with a mortality rate approaching 50% worldwide (1). Sepsis is predominantly a consequence of multiple organ failure, of which myocardial dysfunction is recognized manifestation (2–4). Endotoxin-induced cardiomyocyte apoptosis and the inflammatory response in the cardiovascular system leads to a series of pathophysiological injuries, which significantly increase the mortality rate in patients with sepsis (5–7). Previous studies have demonstrated that the lipopolysaccharide (LPS) bacterial endotoxin reduces contractility and significantly induces the expression of tumor necrosis factor (TNF)-α in cardiomyocytes by binding to toll-like receptor-4 (TLR-4) (8–10). This process activates nuclear factor-κB (NF-κB), which is an important signal integrator controlling the production of pro-inflammatory mediators (11–13). The increased production of numerous inflammatory cytokines, including TNF-α, interleukin (IL)-6, IL-1β, interferon (IFN)γ and intercellular adhesion molecule (ICAM)-1, represses cardiac function directly and indirectly (14). TNF-α is a major pro-inflammatory cytokine, which mediates the signs and symptoms of sepsis and shock (15). In addition, TNF-α-induced apoptotic responses are triggered by the binding of death-receptor ligands to TNF-α receptor 1 (TNF-R1), which is involved in the pathogenesis of cardiac diseases (16).

Estrogen receptors (ERs) are important in preventing endotoxin-induced cardiac dysfunction (16). Notably, clinical studies of patients with sepsis indicate that the mortality rates and expression levels of TNF-α are lower in females compared with males (7,18,19). Estrogen replacement therapy reduces the incidence of heart disease following the menopause (20). In previous years, considerable attention has been paid to identifying natural phytoestrogens, which are plant-derived, polyphenolic, non-steroidal compounds used in preventing and treating cardiovascular diseases (21). *Panax notoginseng*, termed as ‘sanchi’ or ‘san qi’ in Chinese, has been used to prevent and manage cardiovascular disease in China for several years (22). Notoginsenoside R1 (NG-R1), a phytoestrogen, is beleived to be the predominant ingredient of *Panax notoginseng*, which promotes cardiovascular activity (23,24). However, the effects of NG-R1 on cardiomyocytes, and the precise cellular/molecular mechanisms, remain to be elucidated. The present study demonstrated that NG-R1 inhibited the LPS-induced expression of inflammatory cytokines and cell apoptosis in H9c2 cardiomyocytes. As the molecular structure of NG-R1 aglycone is similar to that of estradiol (22), the present study further evaluated whether its cardioprotective effects were dependent on ERs.

## Materials and methods

### Materials

NG-R1 was purchased from Shanghai Winherb Medical S&T Development Co., Ltd. (Shanghai, China). All the tissue culture materials were purchased from Gibco Life Technologies (Grand Island, NY, USA). All antibodies were purchased from Santa Cruz Biotechnology, Inc. (Santa Cruz, CA, USA) and all other chemicals were purchased from Sigma-Aldrich (St. Louis, MO, USA). The endotoxin-free materials used included Dulbecco’s modified Eagle’s medium (DMEM; GE Healthcare Life Sciences, Logan, UT, USA) supplemented with 10% (v/v) fetal bovine serum (FBS; Gibco Life Technologies) and 1% (v/v) penicillin/streptomycin (Gibco Life Technologies). All the investigations performed in the present study were approved by the Ethics Committee of The Second Affiliated Hospital of Harbin Medical University (Heilongjiang, China).

### Cell culture and treatments

H9c2 cardiomyocytes were obtained from the Cell Bank of the Chinese Academy of Sciences (Shanghai, China) and were maintained in DMEM with 4.5 mg/l glucose, supplemented with 10% (v/v) FBS and 1% penicillin/streptomycin (v/v), at 37°C in a humidified atmosphere containing 5% CO_2_. The H9c2 cardiomyocytes (1−10×10^5^) were treated with either the vehicle (0.1% dimethyl sulfoxide; DMSO) or NG-R1 (0, 5, 10, 25 and 50 *μ*M), in the presence or absence of 20 *μ*g/ml LPS. Following pretreatment for 1 h at 37°C with or without NG-R1, the cells were exposed to 20 *μ*g/ml LPS for 24 h at 37°C. In separate experiments, the cells were pretreated with the ICI 182780 (ICI) non-selective ER antagonist, the methyl-piperidino-pyrazole (MPP) selective ERα antagonist, or the selective NF-κB antagonist, PDTC, for 30 min, prior to treatment with either the vehicle (0.1% DMSO) or NG-R1 (25 *μ*M) to examine the effects of ERα and NF-κB in mediating the anti-inflammatory and anti-apoptotic effects of NG-R1.

### Assessment of cell viability and apoptosis

The cell viability was determined using a 3-(4,5-dimethylthiazol-2-yl)-2,5-diphenyl tetrazolium bromide assay, as described previously (25). Cell apoptosis was determined by terminal deoxynucleotidyl transferase dUTP nick end labeling (TUNEL) using an *in situ* cell death detection kit and fluorescein (Roche Applied Science, Quebec, Canada), as described previously (25). Samples were visualized using a BZ-900 fluorescence microscope (Keyence, Osaka, Japan) and Image-Pro Plus software, version 5.0 (Media Cybernetics, Inc., Rockville, MD, USA). A total of 10–20 randomly selected fields were visualized.

### Caspase-3 activity assay

The activity of caspase-3 was measured using the Caspase-3 Fluorometric Assay kit [containing glucose assay buffer, glucose probe (in DMSO), glucose enzyme mix (lyophilized) and glucose standard (100 nmol/ml); catalog no. K105–200; BioVision, Mountain View, CA, USA), according to the manufacturer’s instructions. Each sample in each well of the 96-well plate was filled with 100 *μ*l mixture, including 50 *μ*l resuspended cells in Cell Lysis Buffer, 50 *μ*l 2X Reaction buffer (containing 10 mM final concentration DTT) and 5 *μ*l 1 mM DEVD-AFC substrate (50 *μ*M final concentration). The samples were read using a Fluoroskan Ascent FL fluorometer (Thermo Fisher Scientific, Waltham, MA, USA) with an excitation wavelength of 400 nm and an emission wavelength of 505 nm. The results were expressed as the fold-change compared with the control.

### Reverse transcription-quantitative polymerase chain reaction (RT-qPCR)

The total RNA was extracted using TRIzol reagent (Invitrogen Life Technologies, Carlsbad, CA, USA). An aliquot of the total RNA (~2 *μ*g) was reverse transcribed using a SuperScript First-Strand Synthesis system (Invitrogen Life Technologies.). The resulting cDNA was synthesized from the isolated RNA, and the cycle time values were obtained by RT-qPCR using Power SYBR Green PCR Master mix (Applied Biosystems, Foster City, California, USA) and an iQ5 Real-Time PCR Detection system and analytical software (CFX Manager 2.1; Bio-Rad Laboratories, Inc., Hercules, CA, USA), as described previously (25). The PCR cycling conditions were as follows: Amplification at 95°C for 10 min, followed by 40 cycles of 95°C for 30 sec, 59°C for 30 sec and 72°C for 30 sec. Thermal cycling started with 10 min denaturation at 95°C, 40 cycles of denaturation at 95° C for 15 sec and combined primer annealing/elongation at 60° for 1 min. Each sample was run in triplicate. The primers (BBI Life Sciences Corp., Shanghai, China) were designed using Applied Biosystems Primer Express software (version 2.0) and are shown in Table I. The mRNA expression levels were normalized against GAPDH, and the relative mRNA expression levels are expressed using arbitrary units, with the value of the control group defined as one.

### Western blot analysis

The cell lysate preparation and western blot analysis were performed, as described previously (25), using a western blot kit (BBI Life Science Corp.) according to the manufacturer’s instructions. Cell lysates were subjected to SDS-PAGE (including a 5% stacking gel and a 10% separating gel; Sigma-Aldrich) and transferred to nitrocellulose membranes Beyotime Institute of Biotechnology, Haimen, China). Subsequent to transferring, blots were blocked with 5% milk for 1 h at 37°C. The membranes were probed with the following antibodies: Primary rabbit polyclonal anti-GAPDH (1:200; sc-25778) at 4°C for 72 h as a loading control, mouse monoclonal ERα (1:500; sc-73479), mouse monoclonal ERβ (1:500; sc-390243), mouse monoclonal p-p65 (1:200; sc-166748), rabbit polyclonal total p65 (1:200; sc-372) and mouse monoclonal I-κBα (1:200; sc-373893) at 4°C for 24 h, and horseradish peroxidase-conjugated secondary antibodies (1:5,000; goat anti-mouse IgG-HRP, sc-2005, and goat anti-rabbit IgG-HRP, sc-2004) at room temperature for 30 min. The membranes were washed with Tris-buffered saline with Tween 20 for 10 min three times following incubation with the antibodies. The protein concentration was determined using a Bio-Rad DC Protein Determination kit (Bio-Rad Laboratories, Inc.), with bovine serum albumin as the standard. The immunoblots were developed using an enhanced chemilluminescence kit (GE Healthcare, Little Chalfont, UK). The signals were quantified by Quantity-One software (version 4.62; Bio-Rad Laboratories, Inc.) and the results from each experimental group are expressed as the relative integrated intensity compared with the control.

### Indirect immunofluorescence assays

The H9c2 cardiomyocytes were cultured on Lab-Tek chamber slides (Nalge Nunc International, Naperville, IL, USA) and were fixed using cold 4% methanol at −20°C for 3 min. Indirect immunofluorescence assays were performed, as described previously (26). Briefly, the cells (1−10×10^5^) were treated with 0.3% Triton X-100 in phosphate-buffered saline (PBS) for 15 min at room temperature, to increase permeability. Following blocking with 10% normal goat serum in PBS at room temperature for 1 h, the cell monolayers were screened using a standard indirect immunofluorescence staining procedure, with polyclonal antibodies against the p65 subunit of NF-κB (1:200) and a fluorescein isothiocyanate-labeled anti-rabbit antibody (1:200). The nuclei were stained using 10 *μ*g/ml 4′,6-diamidino-2-phenylindole (Sigma-Aldrich). The negative controls were incubated with preimmune rabbit sera rather than primary antibodies.

### Statistical analysis

The data are expressed as the mean ± standard error of the mean. The significance of the differences between means were assessed using Student’s t-test. A one-way analysis of variance with Bonferroni corrections was used to determine the significance for multiple comparisons. P<0.05 was considered to indicate a statistically significant difference. Statistical calculations were performed using SPSS 11.0 software (SPSS, Inc., Chicago, IL, USA).

## Results

### Inhibition of LPS-induced H9c2 cell death by NG-R1 is mediated by ERα

**Figure 1:**
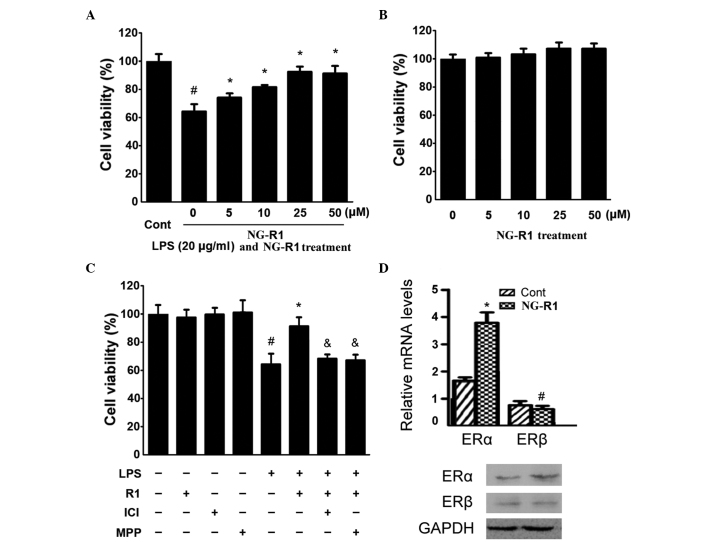
Effects of NG-R1 on the viability of H9c2 cardiomyocytes and ER isoforms. (A) H9c2 cells were treated with the indicated concentrations of NG-R1 (0–50 *μ*M) for 1 h, followed by treatment with LPS (20 *μ*g/ml) for 24 h or (B) with the indicated concentrations (0–50 *μ*M) of NG-R1 for 24 h, and cell viability was determined using an MTT assay expressed as a percentage of the control (n=8 per group; ^#^P<0.05, vs. cells treated with NG-R1 only). (C) Cell viability was determined using an MTT assay and the effects of the ICI and MPP ERα antagonists on H9c2 cardiomyocyte viability were assessed (^#^P<0.05, vs. cells treated without LPS). (D) Reverse transcription-quantitative polymerase chain reaction and immunoblotting revealed that NG-R1 selectively increased the expression of ERα. The results are expressed as the mean ± standard error of the mean. MTT, 3-(4,5-dimethylthiazol-2-yl)-2,5-diphenyl tetrazolium bromide; NG-R1, notoginsenoside R1; Cont, vehicle (0.1% dimethyl sulfoxide); LPS, lipopolysaccharide; R1, NG-R1; ICI, ICI 182780; MPP, methyl-piperidino-pyrazole; ER, estrogen receptor.

Following incubation with various concentrations of LPS (0–20 *μ*g/ml) for 24 h, a significant, dose-dependent reduction in cell viability was observed (Fig. 1). Therefore, a dose of 20 *μ*g/ml was selected for subsequent experiments. As shown in Fig. 1A, LPS (20 *μ*g/ml) significantly reduced cell viability by ~35%, whereas pretreatment with 5, 10, and 25 *μ*M NG-R1 maintained cell viability at ~73, 81 and 92%, respectively. By contrast, the viability of the H9c2 cells remained unaltered following treatment with NG-R1 alone (Fig. 1B). These results suggested that NG-R1 inhibited LPS-induced cell death in a dose-dependent manner. Since a higher concentration of NG-R1 (50 *μ*M) demonstrated no additional benefit on cell viability, a dose of 25 *μ*M was selected for subsequent experiments.

To detect whether the inhibitory effect of NG-R1 on LPS-induced H9c2 cell death is mediated by ER, an ERα antagonists was used to pretreat the H9c2 cells prior to treatment with LPS and NG-R1. As shown in Fig. 1C, the effects of NG-R1 on H9c2 cell viability were attenuated by 30 min pretreatment with ICI, a non-selective ERα antagonist, or MPP, a selective ERα antagonist, prior to treatment with NG-R1 (25 *μ*M) followed by LPS (20 *μ*g/ml). Notably, NG-R1, ICI or MPP alone exerted no effects on cell viability. These results suggested that NG-R1 inhibited LPS-induced cell death in an ER-dependent manner. The effect of NG-R1 on the expression of ER was also determined. As shown in Fig. 1D, increases in the mRNA and protein expression levels of ERα in NG-R1-treated cardiomyocytes were observed. However, the expression of ERβ remained unaltered. Taken together, these results demonstrated that NG-R1 acted through ERα.

### Inhibition of LPS-induced H9c2 cell apoptosis by NG-R1 is mediated by ERα

**Figure 2:**
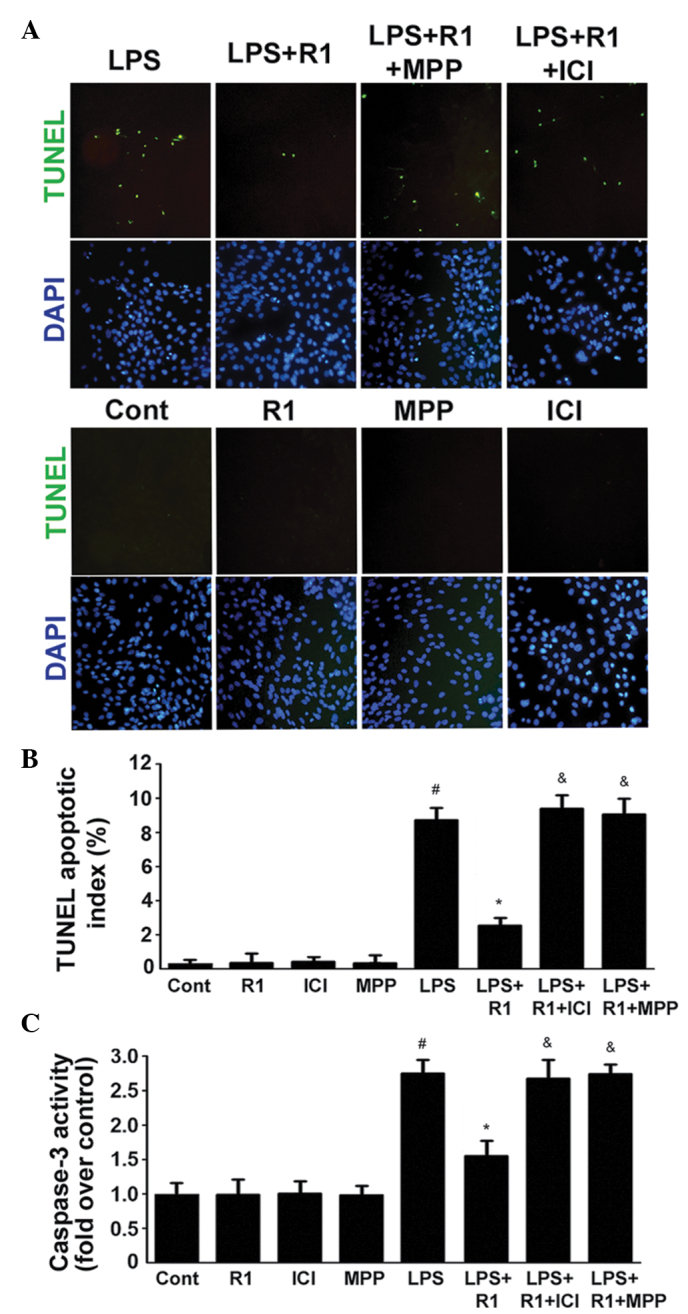
Effects of LPS, NG-R1 and/or ER antagonists on the apoptosis of H9c2 cardiomyocytes. (A) ERα mediates the effects of NG-R1 on endotoxin-induced inflammatory and apoptotic responses in H9c2 cardiomyocytes. Cells were pre-incubated with ICI or MPP for 30 min prior to treatment with or without NG-R1 (25 *μ*M) for 1 h, followed by LPS (20 *μ*g/ml) for 24 h. The cells were subsequently fixed and subjected to TUNEL and DAPI staining (magnification, ×200). (B) TUNEL apoptotic index was determined by calculating the ratio of TUNEL-positive cells to total cells. (C) Caspase-3 activity was measured using a fluorometric assay, and expressed as the fold-change compared with the control. The data are presented as the mean ± standard error of the mean (n=8 per group; ^#^P<0.05, vs. Cont; ^*^P<0.05, vs. LPS treatment; ^&^P<0.05, vs. NG-R1 and LPS co-treatment). TUNEL, terminal deoxynucleotidyl transferase dUTP nick end labeling; DAPI, 4′,6-diamidino-2-phenylindole; NG-R1, notoginsenoside R1; Cont, vehicle (0.1% dimethyl sulfoxide); LPS, lipopolysaccharide; R1, NG-R1; ICI, ICI 182780; MPP, methyl-piperidino-pyrazole; ER, estrogen receptor.

The apoptotic index and the activity of caspase-3 were examined in the H9c2 cardiomyocytes (Fig. 2). In cells treated with LPS (20 *μ*g/ml), DNA fragmentation was observed following treatment for 24 h (Fig. 2A and B). This finding confirmed the data shown in Fig. 2C, demonstrating that caspase-3 was activated following treatment with LPS. By contrast, treatment with NG-R1 (25 *μ*M) effectively ameliorated the LPS-induced DNA fragmentation and activation of caspase-3. In addition, the effects of NG-R1 on the apoptotic index and the activity of caspase-3 were attenuated following 30 min pretreatment with ICI or MPP, prior to treatment with NG-R1 (25 *μ*M) and subsequently LPS (20 *μ*g/ml). NG-R1, ICI or MPP alone exerted no effects on these processes.

### ERa-mediated inhibition of NF-κB contributes to the inhibitory effect of NG-R1 on LPS-induced apoptosis of H9c2 cells

**Figure 3:**
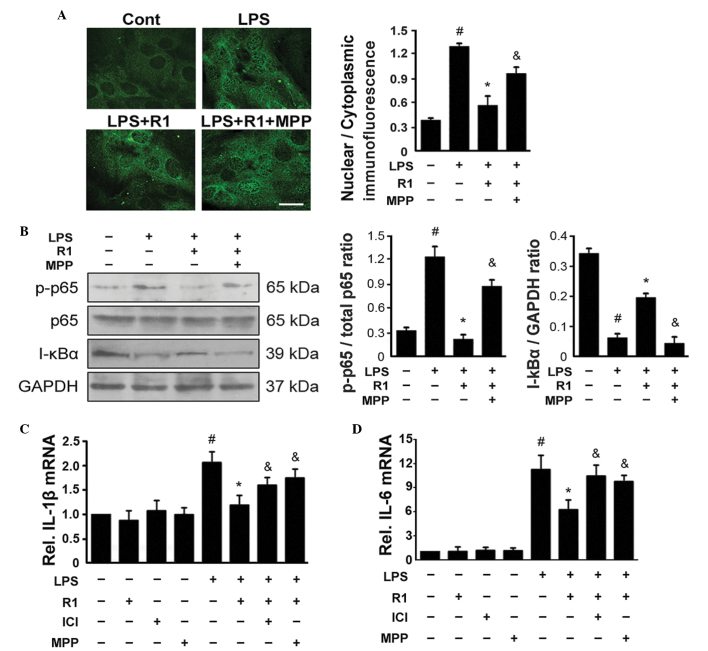
NG-R1-mediated inhibition of LPS-mediated activation of NF-κB is dependent on ERα. (A) Cells were pre-incubated with MPP, a selective ERα antagonist, for 30 min prior to treatment with or without NG-R1 (25 *μ*M) for 1 h, followed by LPS (20 *μg*/ml) for 24 h. Representative images of indirect immunofluorescence for the p65 subunit of NF-κB in H9c2 cells are shown (scale bar=10 *μ*m). The mean density ratios of nuclear/cytoplasmic immunofluorescence were analyzed. (B) Lysates were prepared from the H9c2 cells and the immunoblots were probed for p-p65, total p65 and I-κBα, and quantitative analyses of the phosphorylation of NF-κB and degradation of I-κBα in the H9c2 cells was performed. GAPDH was used as a loading control in all western blotting experiments. (C and D) Expression levels of IL-1β and IL-6 were measured by reverse transcription-quantitative polymerase chain reaction (n=6 per group; ^#^P<0.05, vs. Cont; *P<0.05, vs. LPS-treatment; ^&^P<0.05, vs. NG-R1 and LPS co-treatment. NG-R1, notoginsenoside R1; Cont, vehicle (0.1% dimethyl sulfoxide); LPS, lipopolysaccharide; R1, NG-R1; ICI, ICI 182780; MPP, methyl-piperidino-pyrazole; ER, estrogen receptor; p-, phosphorylated; IL, interleukin.

**Figure 4:**
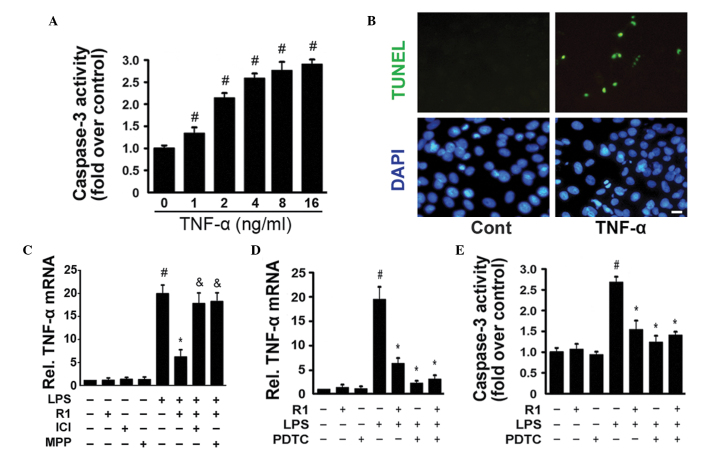
TNFα stimulates the activation of caspase-3 and apoptosis in H9c2 cardiomyocytes. (A) H9c2 cells were cultured with the indicated concentrations (0–16 ng/ml) of TNF-α for 24 h. The activity of caspase-3 was measured using a fluorometric assay and expressed as the fold-change compared with the control (n=6 per group; ^#^P<0.05, vs. Cont). (B) Cells were exposed to TNF-α (16 ng/ml) for 24 h and were stained using TUNEL and DAPI staining (scale bar=10 *μ*m). (C) mRNA expression of TNF-α was measured by reverse transcription-quantitative polymerase chain reaction (n=6 per group; ^#^P<0.05, vs. Cont; *P<0.05, vs. LPS-treatment; ^&^P<0.05, vs. NG-R1 and LPS co-treatment. (D) Cells were pre-incubated with PDTC (a specific inhibitor of NF-κB) for 30 min prior to treatment with or without NG-R1 (25 *μ*M) for 1 h, followed by LPS (20 *μg*/ml) for 24 h. The mRNA expression of TNF-α was determined byRT-qPCR (E) Caspase-3 activity was measured using a fluorometric assay and expressed as the fold-change compared with the control (n=6 per group; #P<0.05, vs. Cont; *P<0.05, vs. LPS-treatment; ^&^P<0.05, vs. NG-R1 and LPS co-treatment). The results are expressed as the mean ± standard error of the mean. TUNEL, terminal deoxynucleotidyl transferase dUTP nick end labeling; DAPI, 4′,6-diamidino-2-phenylindole; NG-R1, notoginsenoside R1; Cont, vehicle (0.1% dimethyl sulfoxide); LPS, lipopolysaccharide; R1, NG-R1; ICI, ICI 182780; MPP, methyl-piperidino-pyrazole; TNF, tumor necrosis factor; PDTC, pyrrolidine dithiocarbamate; RT-qPCR, reverse transcription quantitative polymerase chain reaction.

Treatment with LPS resulted in the activation of NF-κB in several types of cells, which was characterized by the nuclear translocation of NF-κB following the phosphorylation of NF-κB p65 and degradation of the NF-κB inhibitor α (I-κBα) (27–29). The present study used immunofluorescence staining of NF-κB p65 and demonstrated that treatment with LPS led to nuclear accumulation of NF-κB in the H9c2 cells (Fig. 3A). Western blotting revealed that treatment with LPS led to the phosphorylation of p65 and degradation of I-κBα (Fig. 3B). By contrast, treatment with NG-R1 (25 *μ*M) reduced the LPS-induced phosphorylation of NF-κB p65, degradation of I-κBα and nuclear localization of NF-κB. These effects were attenuated by pretreatment with MPP 30 min prior to treatment with NG-R1 (25 *μ*M) and subsequently LPS (20 *μ*g/ml). Treatment with either NG-R1 or MPP alone exerted no effects on the phosphorylation of NF-κB p65, degradation of I-κBα or nuclear localization of NF-κB. as shown in Fig. 3C and D, exposure of the H9c2 cells to LPS increased the production of NF-κB target genes, including IL-6 and IL-1β. The expression levels of these genes increased following treatment with LPS, however they were significantly inhibited by additional treatment with NG-R1. The effects of NG-R1 on the expression levels of these NF-κB target genes were attenuated by pretreatment with ICI or MPP 30 min prior to treatment with NG-R1 (25 *μ*M) and subsequently LPS (20 *μ*g/ml). NG-R1, ICI or MPP alone exerted no effects on the expression levels of the NF-κB target genes. These data indicated that NG-R1 inhibited the LPS-induced activation of NF-κB in an ERα-dependent manner. TNF-α (0–16 ng/ml) significantly activated caspase-3 in a dose-dependent manner (Fig. 4A) and TUNEL staining revealed TNF-α-induced myocardial cell apoptosis (Fig. 4B). In the H9c2 cells, NG-R1 inhibited the LPS-induced expression of TNF-α, and this effect was attenuated by treatment with ICI or MMP (Fig. 4C). Similar to the effects of the NF-κB inhibitor, pyrrolidine dithiocarbamate (PDTC), NG-R1 significantly inhibited the LPS-induced expression of TNF-α and the activation of caspase-3 (Fig. 4D and E). NG-R1 alone exerted no effects on the expression of TNF-α or the activation of caspase-3. These data confirmed that the inhibitory effects of NG-R1 on the TNF-α-mediated activation of caspase-3 and apoptosis in H9c2 cells were closely associated with the inactivation of NF-κB.

## Discussion

NG-R1, a phytoestrogen, is believed to be the predominant ingredient in *Panax notoginseng* responsible for its cardiovascular activity. However, the effects of NG-R1 on cardiomyocytes, and its precise cellular/molecular mechanisms, remain to be elucidated. The present study observed for the first time, to the best of our knowledge, that NG-R1 significantly attenuated endotoxin-induced inflammatory and apoptotic responses in H9c2 cardiomyocytes. Furthermore, the cardioprotective effects of NG-R1 were dependent on the activation of ERα and the inactivation of NF-κB in these cells.

Septic shock, resulting from host stimulation of inflammatory cytokines, causes cardiac dysfunction by suppressing myocardial contractility, which significantly increases mortality rates in patients with sepsis (27). Bacterial LPS is a potent stimulator of proinflammatory cytokines, including TNF-α, IL-6, IL-1β, IFNγ and ICAM-1, in cardiomyocytes (27). The results of the present study demonstrated that NG-R1 increased cell viability and reduced apoptotic damage in cardiomyocytes via the inhibition of a series of proinflammatory cytokines, including TNF-α, IL-6, IL-1β and IFNγ (Figs. 1^3^). NG-R1 also inhibited the activation of NF-κB signaling in cardiomyocytes, as demonstrated by phosphorylation of the p65 subunit of NF-κB and degradation of I-κBα (Fig. 4). In cardiomyocytes, TLR4 specifically recognizes LPS, resulting in the activation of NF-κB, which is an important signal integrator controlling the production of pro-inflammatory mediators (27). Among these mediators, TNF-α, a major proinflammatory cytokine, induces an apoptotic responses by promoting the binding of death-receptor ligands to TNF-R1, subsequently initiating the death-receptor-mediated apoptotic pathway (29). The present study suggested that activation of NF-κB caused the upregulation of TNF-α in myocardial cells, which directly contributed to cardiac apoptosis, as demonstrated by the increased quantities of TUNEL-positive cells and the activation of caspase-3 in cardiomyocytes following stimulation with TNF-α (Fig. 2). In addition, the NF-κB activation inhibitor, PDTC, partially inhibited the production of TNF-α and LPS-mediated activation of caspase-3 in myocardial cells (Figs. 4C and D). These results confirmed those of previous studies demonstrating that the induction of myocardial inflammatory cytokines, including TNF-α, IL-1β, and IL-6, is critical for activation of caspase in endotoxemic models (19,30). The data also confirmed previous reports that LPS-induced TNF-α is responsible for myocardial cell apoptosis via the NF-κB signaling pathway (29).

Estrogen and ERs are implicated in the cellular survival of cardiomyocytes (31). The 17β-estradiol ERα agonist reduces pathological cardiac hypertrophy and heart failure (32). To investigate the direct effects of LPS and NG-R1 on cardiomyocytes, and the role of ERα in this process, the present study used pharmacological inhibitors of ERα, ICI and MPP The results revealed that the ability of NG-R1 to inhibit apoptotic and inflammatory responses was dependent on the activation of ERα. These findings were supported by the observation that pharmacologic inhibition of ERα, using ICI or MPP, eliminated the protective effect of NG-R1 against LPS-induced cell death, proinflammatory cytokine production and activation of NF-κB in cardiomyocytes (Figs. 1^4^). In addition, NG-R1 increased the mRNA and protein expression levels of ERα in the NG-R1-treated H9c2 cardiomyocytes, but, it did not alter the expression of ERβ (Fig. 1C and D). This finding was in accordance with previous reports, which suggested that the activation of ERα in cardiomyocytes attenuates the LPS-induced expression of TNF-α and myocardial cell apoptosis (29).

In the present study, pretreatment with NG-R1 caused the activation of ERα (Fig. 1). There is a missing link between the NG-R1-mediated activation of ERα and the NG-R1-mediated inhibition of cell apoptosis, decreased caspase-3 activity, or NG-R1-mediated attenuation of the inflammatory response (downregulated NF-κB activation and reduced cytokine expression_. It is well-documented that ERα activates the phosphoinositide 3-kinase (PI3K)/Akt and mitogen-activated protein kinase (MAPK) signaling pathways, thereby negatively regulating LPS-induced NF-κB-dependent inflammatory responses in several cell types, including cardiomyocytes (32). Therefore, NG-R1 may also inhibit apoptotic and inflammatory responses through the PI3K/Akt and/or MAPK signaling pathways, although further investigation is required.

Another issue to address is that, as an estrogen-like compound, NG-R1 is a tetracyclic triterpenoid saponin with a weak estrogenic effect, and the binding capacity of saponins to ERs is poor *in vivo* (32). Therefore, the significant protective effects of NG-R1 in the present study are not limited to its estrogenic properties. Previous studies have demonstrated that pretreatment with NG-R1 may also act on the PI3K/Akt and reactive oxygen species (ROS)/extracellular signal-regulated kinase signaling pathways and directly scavenge ROS (22). In addition, NG-R1 has exhibited other multifunctional functions in cardioprotection, including attenuating the LPS-induced activation of the coagulation system, reducing fibrinolytic capacity and inhibiting neutrophil/leukocyte infiltration and inflammatory reactions (24).

In conclusion, the present study revealed that pretreatment with NG-R1 improved cell viability, inhibited inflammatory cytokine production and attenuated the LPS-induced activation of NF-κB in cardiomyocytes. The activation of ERα and inhibition of the NF-κB signaling pathway in cardiomyocytes is, therefore, important for the cardioprotective effects of NG-R1. In addition to our previous studies demonstrating that NG-R1 attenuates cardiac dysfunction in the myocardium of endotoxemic mice (33,34), the present study suggested that NG-R1 exerts direct anti-inflammatory effects on cardiomyocytes. Thus, NG-R1 represents a potent reagent for the treatment of myocardial inflammation during septic shock.

## Figures and Tables

**Table I:** Primers used for reverse transcription-quantitative polymerase chain reaction.

Target gene	Forward sequence (5′–3′)	Base pairs	Reverse sequence (5′–3′)	Base pairs
ER-α	TCCCCAACACCATCTGAGAACT	22	CGTTTCAGGGATTCGCAGAA	20
ER-β	TCAGGAAAAGGAATATGGCATGT	23	TTTTATGGCCACACAGTCCTACA	23
TNF-α	CATCTTCTCAAAATTCGAGTGACAA	25	TGGGAGTAGACAAGGTACAACCC	23
IL-1β	CAACCAACAAGTGATATTCTCCATG	25	GATCCACACTCTCCAGCTGCA	21
IL-6	GAGGATACCACTCCCAACAGACC	23	AAGTGCATCATCGTTGTTCATACA	24
INFγ	CGCCGCGTCTTGGTTTT	27	GAGTGTGCCTTGGCAGTAACAG	22
GAPDH	AACGACCCCTTCATTGAC	22	TCCACGACATACTCAGCAC	19

ER, estrogen receptor; TNF, tumor necrosis factor; IL, interleukin; INF, interferon.
